# Cavum septum pellucidum: a novel endoscopic approach to the posterior third ventricle

**DOI:** 10.1186/2045-8118-12-S1-P3

**Published:** 2015-09-18

**Authors:** Hasan Asif, Nithish Jayakumar, Peter Heppner

**Affiliations:** 1 Department of Neurosurgery, Starship Children's Hospital, Auckland, New Zealand

## Objectives

To describe a novel endoscopic approach to the posterior third ventricle through a cavum septum pellucidum.

## Design and subjects

Retrospective case review of a 7-year old boy with a tectal plate mass.

## Methods

The clinical and operative notes, operative videos, and imaging were reviewed. A literature search was also performed.

## Results

The patient was referred for neurosurgical opinion due to a 2-week history of gait instability on a background of progressive ataxia and urinary incontinence. MRI showed triventricular hydrocephalus due to a tectal plate mass with thalamic extension.

On examination, his GCS was 15/15 and he demonstrated truncal ataxia, gait instability, and upper limb dysmetria. He was admitted for an endoscopic third ventriculostomy with biopsy.

Operative approach: The patient was anaesthetised and placed supine. Incision and burr hole were over the left coronal suture. A third ventriculostomy was performed for hydrocephalus.

The tumour was then approached through the cavum septum pellucidum. Septal fenestrations allowed easy access to the cavum. Internal cerebral veins were identified and the velum interpositum opened with sharp scissors. The opening was dilated and an endoscope advanced into the posterior third ventricle. The tumour was identified and biopsies obtained.

He had a satisfactory recovery and was discharged 6 days later. Histology showed a low-grade glioma, which remained stable on follow-up MRI.

## Conclusions

When present, the cavum septum pellucidum provides a viable alternative route to the posterior third ventricle.

